# Esketamine as an Adjuvant to Ciprofol or Propofol Sedation for Same-Day Bidirectional Endoscopy: Protocol for a Randomized, Double-Blind, Controlled Trial With Factorial Design

**DOI:** 10.3389/fphar.2022.821691

**Published:** 2022-03-18

**Authors:** Yu-qin Long, Chang-dong Feng, Yun-ying Ding, Xiao-mei Feng, Hong Liu, Fu-hai Ji, Ke Peng

**Affiliations:** ^1^ Department of Anesthesiology, First Affiliated Hospital of Soochow University, Suzhou, China; ^2^ Institute of Anesthesiology, Soochow University, Suzhou, China; ^3^ Department of Anesthesiology, University of Utah, Salt Lake City, UT, United States; ^4^ Department of Anesthesiology and Pain Medicine, University of California Davis Health System, Sacramento, CA, United States

**Keywords:** ciprofol, propofol, esketamine, sedation, bidirectional endoscopy

## Abstract

**Background:** Same-day esophagogastroduodenoscopy and colonoscopy procedures under sedation have been increasingly performed. This study aims to assess the effects of esketamine combined with ciprofol (a novel anesthetic/sedative agent) or propofol on respiratory and hemodynamic adverse events in patients undergoing same-day bidirectional endoscopy.

**Methods:** This is a prospective, randomized, double-blind, placebo-controlled, 2 × 2 factorial trial. A total of 180 adult patients scheduled for same-day bidirectional endoscopy under sedation will be randomized, in a 1:1:1:1 ratio, to receive 1 of 4 sedation regimens: 1) ciprofol and esketamine, 2) propofol and esketamine, 3) ciprofol and normal saline placebo, or 4) propofol and normal saline placebo. The primary outcome is a composite of desaturation [peripheral oxygen saturation (SpO_2_) < 95%] and hypotension [mean blood pressure (MBP) < 65 mmHg or decrease in MBP ≥20% of baseline] during the sedation and in the recovery room. Secondary outcomes include episodes of desaturation, severe desaturation (SpO_2_ < 90%), hypotension, severe hypotension (decrease in MBP ≥30% of baseline), bradycardia, postoperative nausea and vomiting, dizziness or headache, hallucination or nightmare, injection pain, pain scores and fatigue scores, endoscopist satisfaction, and patient satisfaction. Data will be analyzed on the modified intention-to-treat basis.

**Discussion:** We hypothesize that esketamine as an adjuvant to ciprofol or propofol sedation would improve cardiorespiratory stability. In addition, the potential interactions between interventions will be explored using the factorial design. The results of this trial will provide evidence for daily practice of sedation regimens for same-day bidirectional endoscopy.

**Clinical Trial Registration:** Chinese Clinical Trials Registry, Identifier ChiCTR2100052523.

## Introduction

Same-day bidirectional endoscopy (esophagogastroduodenoscopy and colonoscopy) has become common, which reduces healthcare cost and enhances decision-making. (Urquhart et al., 2009) In the United States, sedation is provided for more than 98% of endoscopic procedures to alleviate patients’ discomfort and facilitate the implementation of procedures. (Cohen et al., 2006) Recent studies have shown that the esophagogastroduodenoscopy-colonoscopy sequence (esophagogastroduodenoscopy followed by colonoscopy) is the optimal sequence for patients undergoing same-day bidirectional endoscopy under sedation. (Cao et al., 2017; Laoveeravat et al., 2020)

Propofol is the most used sedative agent for gastrointestinal endoscopy. (Zhang et al., 2018) However, adverse events such as over-sedation, desaturation, and hypotension are associated with propofol sedation. Using an adjunct medication (such as fentanyl, ketamine, or lidocaine) during propofol sedation reduced the consumption of propofol and associated adverse events. (Chiang et al., 2013; Forster et al., 2018) Esketamine is an s-enantiomer of ketamine and an antagonist of N-methyl-D-aspartate receptors, providing more potent sedative and analgesic effects and less psychotropic side effects than the racemic ketamine. (Pfenninger et al., 2002) A low dose of esketamine reduced the need for propofol and maintained cardiorespiratory stability in endoscopic procedures. (Eberl et al., 2020) Ciprofol (HSK3486) is a novel anesthetic/sedative agent similar to propofol, with an equivalent efficacy ratio to propofol of 1/4 to 1/5. (Qin et al., 2017; Bian et al., 2021; Li et al., 2021) A recent study suggested that ciprofol sedation during colonoscopy, when compared to propofol, had good efficacy and safety profiles, reduced injection pain, and may decrease respiratory and hemodynamic depression. (Teng et al., 2021) Despite these promising findings, the optimal sedation regimen to facilitate bidirectional endoscopic procedures is still unclear.

This study aims to investigate the effects of esketamine vs. placebo combined with ciprofol or propofol for sedation in patients undergoing same-day bidirectional endoscopy. The primary outcome measure is a composite of desaturation and hypotension assessed during the sedation and in the recovery room. We hypothesize that esketamine as an adjuvant to ciprofol or propofol sedation would lead to a decrease in the incidence of respiratory and hemodynamic adverse events in these patients.

## Methods

### Ethics and Registration

This study will be conducted at The First Affiliated Hospital of Soochow University, Suzhou, Jiangsu, China. The study protocol was approved by the Institutional Review Board at The First Affiliated Hospital of Soochow University on 12 October 2021. This study was registered at the Chinese Clinical Trials Registry (Identifier: ChiCTR2100052523) on 30 October 2021. This study will be conducted in accordance with the Declaration of Helsinki and the International Conference on Harmonisation guidelines for Good Clinical Practice. This protocol strictly follows the reporting guideline of Standard Protocol Items: Recommendations for Interventional Trials (SPIRIT) (Supplementary file 1). (Chan et al., 2013)

### Trial Design

This is a single-center, randomized, double-blind, placebo-controlled, 2 × 2 factorial clinical trial. The 2 × 2 factorial design allows researchers to examine the main effects of two interventions simultaneously and explore possible interaction effects. (Green et al., 2002; Pandis et al., 2014; Haerling Adamson and Prion, 2020) In this trial, the use of adjunct medication has two options: esketamine or normal saline placebo; the selection of sedative also has two options: ciprofol or propofol. Thus, this study consists of the following 4 groups: 1) ciprofol and esketamine, 2) propofol and esketamine, 3) ciprofol and placebo, and 4) propofol and placebo. The main effects comparison will be the treatment effects of esketamine versus placebo as an adjuvant to ciprofol or propofol sedation.

### Trial Status

**FIGURE 1:**
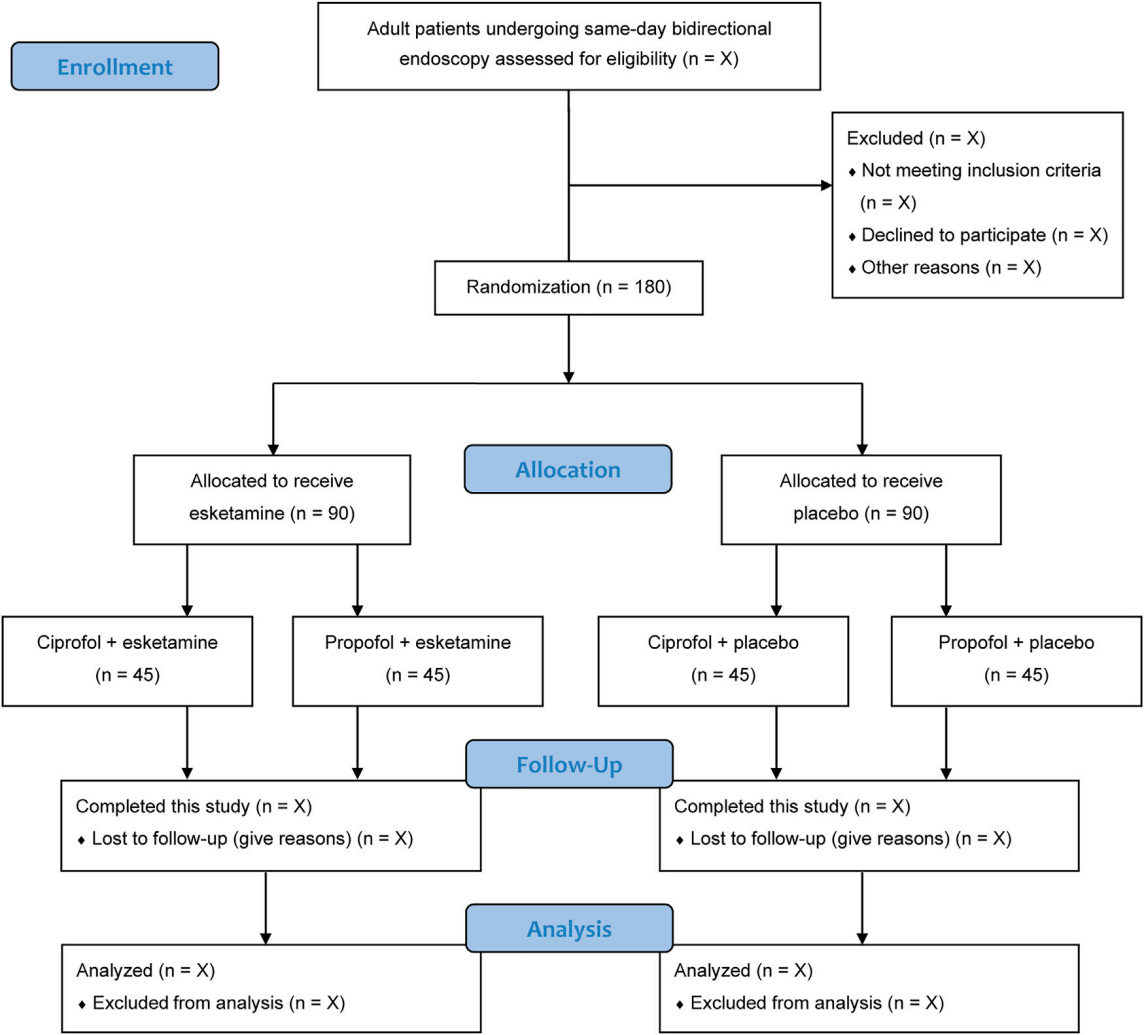
Flow chart of patients.

The current status of this trial is ongoing. The recruitment of participants begins in December 2021. We expect this trial to be completed before October 2022. The flow chart of this trial is represented in Figure 1.

### Eligibility Criteria

An independent investigator who is not involved in the subsequent study screens the admission records for recruitment of eligible patients. Written informed consent will be obtained from each patient. The inclusion criteria are: 1) 18 years or older; 2) American Society of Anesthesiologists (ASA) physical status I to III; and 3) scheduled for same-day bidirectional endoscopy under sedation. The exclusion criteria are: 1) severe cardiovascular or pulmonary diseases; 2) renal or liver dysfunction; 3) neurocognitive or psychiatric disorders; 4) epilepsy; 5) hypersensitivity to study medications; 6) drug or alcohol misuse; or 7) refusal for participation.

### Randomization and Blinding

An independent biostatistician who is not involved in data collection or analysis generates a sequence of random numbers, with an allocation ratio of 1:1:1:1 and block size of 8. Allocation concealment is ensured using sealed opaque envelopes. According to the randomization results, patients will be assigned to 1 of 4 sedation regimen groups (*n* = 45 in each group). Both ciprofol and propofol are emulsions, and both esketamine and normal saline placebo are clear and colorless fluids. To achieve blinding to group assignment among patients, care teams, and outcome evaluators, an independent research nurse distributes the study medications in identical syringes labelled with study numbers only. In addition, as the administration of esketamine can produce sedative effects, esketamine or placebo will be infused immediately after loss of consciousness to avoid interference with blinding.

### Study Interventions

All patients will receive intravenous sedation administered by the same anesthesiologist unaware of patient allocation group. For induction of sedation, an intravenous bolus injection of ciprofol 0.2 mg/kg or propofol 1 mg/kg will be administered. After loss of consciousness, patients will receive intravenous esketamine 0.2 mg/kg or the same volume of placebo. The level of sedation will be assessed using the Ramsay Sedation Scale (RSS, ranging from 1 to 6). (Ramsay et al., 1974) Additional doses of ciprofol 0.06–0.1 mg/kg or propofol 0.3–0.5 mg/kg will be used to achieve an RSS score of 5 (sluggish response to stimulus) or 6 (no response) and to treat discomfort responses (such as body movement, gag, cough, moan, hiccup, or grimaces).

All bidirectional endoscopic procedures will be performed in the esophagogastroduodenoscopy-colonoscopy sequence. (Cao et al., 2017; Laoveeravat et al., 2020) Throughout the study, patients will receive cuff noninvasive blood pressure and pulse oximetry monitoring. Supplemental oxygen at 3 L/min will be given through a nasal catheter. Upon the completion of endoscopic procedures, patients will be transferred to a recovery room. All patients will stay in the recovery room for at least 30 min. A modified Aldrete score of 10 indicates readiness to be discharged. (Laoveeravat et al., 2020) The schedules of patient enrollment, study interventions, and outcome assessments will be in accordance with the SPIRIT statement (Table 1).

**TABLE 1:** Schedule of enrollment, interventions, and assessments.

Timepoint	Study period						
	Enrollment	Allocation	Post-allocation				Close-out
	Anesthesia clinic visit	Prior to sedation	During sedation	Sedation emergence	15 min in recovery room	30 min in recovery room	Hospital discharge
**Enrollment**	—	—	—	—	—	—	—
Eligibility criteria	×	—	—	—	—	—	—
Written informed consent	×	—	—	—	—	—	—
Demographic data	×	—	—	—	—	—	—
Baseline characteristics	×	—	—	—	—	—	—
Randomization	—	×	—	—	—	—	—
Allocation	—	×	—	—	—	—	—
**Interventions**	—		—	—	—	—	—
Ciprofol and esketamine	—	×	—	—	—	—	—
Propofol and esketamine	—	×	—	—	—	—	—
Ciprofol and placebo	—	×	—	—	—	—	—
Propofol and placebo	—	×	—	—	—	—	—
**Assessments**	—	—	—	—	—	—	—
Desaturation events	—	—	×	×	×	×	×
Hypotension events	—	—	×	×	×	×	×
Severe desaturation	—	—	×	×	×	×	×
Severe hypotension	—	—	×	×	×	×	×
Bradycardia	—	—	×	×	×	×	×
Nausea and vomiting	—	—	—	×	×	×	×
Dizziness or headache	—	—	—	×	×	×	×
Hallucination or nightmare	—	—	—	×	×	×	×
Injection pain	—	—	×	—	—	—	—
VAS pain scores	—	—	—	×	×	×	—
VAS fatigue scores	—	—	—	×	×	×	
Endoscopist satisfaction	—	—	×	—	—	—	—
Patient satisfaction	—	—	—	—	—	—	×

According to SPIRIT, 2013 statement of defining standard protocol items for clinical trials.

VAS, visual analog scale.

### Primary Outcome

The primary outcome measure is a composite of desaturation and hypotension assessed during the sedation and in the recovery room. In other words, if a patient experiences either an episode of desaturation or hypotension or both, such an event will be considered as the primary outcome. Desaturation is defined as peripheral oxygen saturation (SpO_2_) < 95%. Hypotension is defined as mean blood pressure (MBP) < 65 mmHg or decrease in MBP ≥20% of baseline value. Patient’s baseline hemodynamic values will be documented when they are comfortably seated in a preoperative waiting room.

### Secondary Outcomes

The secondary outcomes are: 1) the incidence of desaturation; 2) the incidence of severe desaturation (SpO_2_ < 90%); 3) the incidence of hypotension; 4) the incidence of severe hypotension (decrease in MBP ≥30% of baseline value); 5) the incidence of bradycardia (heart rate <50 beats/min); 6) the incidence of postoperative nausea and vomiting (PONV); 7) the occurrence of dizziness or headache; 8) the occurrence of hallucination or nightmare; 9) injection pain; (10) pain scores at emergence and 15 and 30 min in the recovery room; 11) fatigue scores at emergence and 15 and 30 min in the recovery room; 12) endoscopist satisfaction; and 13) patient satisfaction. Pain or fatigue scores will be measured using the visual analog scale (VAS, ranging from 0 to 10), with higher scores indicating greater pain or fatigue. Satisfaction will be assessed using a 5-point Likert scoring system, ranging from 1 (very dissatisfied) to 5 (highly satified). (Eberl et al., 2020)

### Data Collection

Demographic data [including age, sex, race, height, weight, and body mass index (BMI)] and baseline characteristics (including comorbidities, preoperative medications, baseline MAP and HR values, and ASA status) will be collected before the procedures. The primary and secondary outcome measures and other perioperative data (total dose of ciprofol or propofol, duration of endoscopy, and time to emergence) will be documented by a trained independent investigator blinded to group assignment. All raw data will be collected in the Case Report Forms. The lead investigator is responsible for data completeness and accuracy. The de-identified data will be stored in the electronic database (https://www.91trial.com) and monitored by an independent Data Monitoring Committee (DMC).

### Safety Monitoring

Propofol has been wide used for sedation in various clinical settings, and emerging literature have shown the use of ciprofol or esketamine for patients undergoing gastrointestinal endoscopic procedures. (Wang et al., 2019; Eberl et al., 2020; Teng et al., 2021) Thus, serious adverse events associated with interventions in this study are less likely to occur. However, in case of a severe adverse event, the attending anesthesiologist will provide immediate clinical treatment, and such an event should be reported to the principal investigator and the Institutional Review Board. They will decide whether the unmasking process of group allocation should be done.

### Sample Size Calculation

The composite incidence of desaturation and hypotension events is reported to be approximately 40% in patients undergoing bidirectional endoscopy with propofol sedation. (Chiang et al., 2013) We hypothesize that the use of esketamine would reduce this incidence to 20%. This trial is powered to detect differences between patients who receive esketamine and placebo. With *α* = 0.05 and power = 80%, 160 patients will be needed per group of esketamine or placebo, with ciprofol or propofol. To account for a potential withdrawal rate of 10%, we plan to enroll a total of 180 patients in this study (n = 45 in each group). The sample size calculation is performed using the PASS software (version 11.0.7, NCSS, LCC, Kaysville, UT, United States).

### Statistical Analysis

Data will be presented as means and standard deviations, medians and interquartile ranges, or numbers and percentages, depending on data type and distribution. Normality will be checked using the Shapiro-Wilk test. Continuous data will be assessed using the unpaired *t* test, repeated measures analysis of variance, or Wilcoxon rank sum test, as appropriate. Categorical data will be assessed using the chi-squared test or Fisher’s exact test.

For the primary and secondary outcomes, mean differences or odds ratios with 95% confidence intervals between groups will be analyzed. Subgroup analyses of the primary outcome will be conducted according to age (≤60 years vs. > 60 years) and BMI (≤28 kg/m^2^ vs. > 28 kg/m^2^). For the secondary outcomes, corrections for multiple comparisons will be conducted with calculation of the false-discovery rate using the Benjamini–Hochberg method. As this is a trial with factorial design, a further analysis will be performed to assess the interaction between groups using a regression model. In this model, the primary outcome (a composite of desaturation and hypotension) is the outcome variable, with an interaction term between the sedative (ciprofol or propofol) and adjuvant groups (esketamine or placebo).

Data will be analyzed on a modified intention-to-treat basis, which includes all randomized patients receiving at least one dose of the study medication. As missing data is expected to be uncommon in this study, we do not plan to perform data imputation. All analyses will be performed using the SPSS software (version 19.0; IBM SPSS, Chicago, IL, United States). A two-sided *p* value <0.05 will be considered statistically significant.

## Discussion

As far as we know, this is the first randomized clinical trial powered to investigate the use of esketamine as an adjuvant to ciprofol or propofol sedation on respiratory and hemodynamic adverse events in patients undergoing same-day bidirectional endoscopy. In addition, we will analyze the incidence of bradycardia, PONV, dizziness, headache, hallucination, nightmare, injection pain, pain scores and fatigue scores up to 30 min in the recovery room, endoscopist satisfaction, and patient satisfaction. Moreover, the interactions between the adjunct medication (esketamine) and the sedatives (ciprofol or propofol) will be explored based on the factorial design.

According to a recent national survey, the sedation rate for gastrointestinal endoscopy was approximately 50% in China, which is much lower than that in the United States and Europe. (Zhou et al., 2021) With the increasingly performed endoscopic procedures under sedation, the risk for complications such as cardiorespiratory depression should be cautioned. Despite many benefits of same-day bidirectional endoscopy, a prolonged duration of sedation is needed in this procedure than in gastroscopy or colonoscopy alone, which increases the risk for respiratory and hemodynamic adverse events. Currently, there are no recommendations on the optimal sedative regimen for gastrointestinal endoscopy. To examine this question, this 2 × 2 factorial trial will use esketamine or placebo in combination with ciprofol or propofol as study interventions, aiming to provide evidence for daily practice of sedation regimens for patients undergoing same-day bidirectional endoscopy.

Propofol has been widely used for safe and controlled sedation/anesthesia around the world. (Walsh, 2018) However, propofol can lead to dose-dependent depression of blood pressure and respiration. In addition, the incidence of injection pain induced by propofol administration was 25–74%. (Abad-Santos et al., 2003) Ciprofol is a new 2,6-disubstituted phenol derivative and a close analog of propofol. (Qin et al., 2017; Bian et al., 2021) Compared with propofol, the advantages of ciprofol are as follows: 1) a stronger affinity for the gamma-aminobutyric acid-A receptors and 4–5 times more potent than propofol; 2) a stable respiratory profile and less inhibition of cardiac function; 3) less lipid exposure with 1% lipid emulsion; and 4) reduced or eliminated injection pain. (Hu et al., 2021; Li et al., 2021; Teng et al., 2021) Therefore, ciprofol may be a better alternative to propofol when used for sedation and general anesthesia induction.

As ciprofol and esketamine are two novel drugs, the ethical situation should be clarified. To date, there is no approval from the Food and Drug Administration (FDA) or European Medicines Agency (EMA) for the use of ciprofol. A multicenter randomized phase III clinical trial investigating ciprofol (HSK3486) for anesthesia induction in adult surgical patients is ongoing in the United States (NCT04711837). Esketamine has been approved for the treatment of depression only by the FDA and for general anesthesia by the EMA. In China, the National Medical Products Administration (NMPA) has approved the use of ciprofol for sedation and induction of anesthesia (approval no. H20200013) and the use of esketamine for perioperative sedation and analgesia (approval no. H20193336). This study will be conducted at The First Affiliated Hospital of Soochow University, Suzhou, China. We will conform to the Declaration of Helsinki, the Good Clinical Practice guidelines, and the NMPA approvals. All these information have been explicitly stated in the informed consents for patients.

There are several limitations. First, based on the sample size calculation, we plan to include a total of 180 patients into 4 sedation regimen groups. This is a relatively small sample size. Second, this study is powered to show the effects of esketamine on cardiorespiratory stability during ciprofol or propofol sedation; thus, the testing for the interactions between esketamine and the two sedative agents can only be interpreted as exploratory. (Haerling Adamson and Prion, 2020) If detecting an interaction is of specific interest, the trial should be designed with four times the current number of patients. (Green et al., 2002; Haerling Adamson and Prion, 2020) Finally, because this is a single center study, the results must be further confirmed in a larger multicenter trial.

## Conclusion

This randomized, double-blind, placebo-controlled, 2 × 2 factorial trial aims to determine the role of esketamine vs. normal saline placebo as an adjuvant to ciprofol or propofol on the incidence of desaturation and hypotension during same-day bidirectional endoscopy. The implementation of this trial and reporting of the findings will be in accordance with the Consolidated Standards of Reporting Trials guideline. (Moher et al., 2010) We expect that the use of esketamine in combination with ciprofol or propofol sedation would improve respiratory and hemodynamic stability in patients undergoing same-day bidirectional endoscopy.

## Data Availability

The original contributions presented in the study are included in the article/Supplementary Material, further inquiries can be directed to the corresponding authors.
